# Law vs. Justice in the Practice of Medicine and Surgery in Texas

**Published:** 1894-05

**Authors:** James Kennedy

**Affiliations:** Professor of Pharmacy and Dean of the Faculty of the School of Pharmacy of the University of Texas; Galveston


For Texas Medical Journal.

### LAW vs. JUSTICE IN THE PRACTICE OF MEDICINE AND SURGERY IN TEXAS.

BY JAMES KENNEDY, PH. G., M. D.,
Professor of Pharmacy and Dean of the Faculty of the School of Pharmacy of the University of Texas.

HAVING just emerged from the labyrinths of law as applied to the collection of fees for professional services, and obtained a verdict more remarkable than profitable, I have deemed it of sufficient importance and interest to the medical profession of the State of Texas, to bring this subject before you at this time. As nearly everything in law is governed by precedent, you will readily understand how great will be the influence of the judgment only recently rendered in cases of this kind that may come up for adjudication in the future.

I herewith present the facts in the case and its course through the tribunals of law:

In June, 1891, a gentleman undertook the construction of a building without the aid of either architect or contractor. When the construction had progressed to a little beyond the first story, the walls collapsed with the result of killing one man outright and seriously injuring several others. Among those who came under my care was a man who had sustained a comminuted fracture of the femur. In addition to this injury he received many severe contusions on different portions of his body. The one over the sacrum resulted in the formation of an abscess which contributed much to the tediousness of the case. The treatment involved a resection of the thigh and later operation for necrosis of the shaft. The resection was reported in the *New York Medical Record* in detail, so I will not burden you with a repetition of the troublesome features of the case. The patient was under my care for seven months, and required attention daily during this period.

When the patient was placed under my care I was instructed to spare neither pains nor expense, but implored to save his life, and was assured that I would be liberally compensated for my services and that no limit would be placed upon my charges, etc., etc. (This statement probably sounds familiar to many.)

When the patient was discharged the bill was sent to the owner of the building, who, realizing his culpability, had agreed to pay all expenses attached to the treatment of the unfortunates who were injured, but, instead of keeping his promise, very coolly referred me to his lawyer, which functionary, instead of settling the account, with greater coolness than his client offered $200 in payment of my services. This I very promptly declined to accept, for the reason that I considered my services in the case to be worth, at a reasonably low estimate, at least $1200.

The bill was turned over to my attorney, with instructions to sue if necessary. Suit was promptly brought and after some months the case came up for hearing. A postponement was secured on the grounds that certain important witnesses were absent by whom defendant expected to prove that the charges were exorbitant.

The next time that the case was called the defense set up the plea, that, inasmuch as the plaintiff was not registered according to law, he had no standing as a physician in the eyes of the law, and, therefore, was not entitled under the law to collect for his services.

This suit was brought in the District Court, and the judge agreeing with the attorney, or the defendant, rendered a verdict in accordance with this interpretation of the law.

I was not allowed to introduce evidence to prove that I was a graduate of a reputable medical college, and that I had used every reasonable endeavor to register; that I had only failed to comply with the law because the officers of the law whose duty it was to do so, had failed to provide the means.

The present medical law provides for the establishment of a Board of Medical Examiners for each judicial district. In the opinion of my attorney who carried my case up to the higher court (Court of Civil Appeals) the law is nnconstitutional, for the reason that it is in conflict with the Constitution of the State.

I copy the following from the brief submitted to the court by my attorney:

"Art. 3526 of title 73, Revised Statutes, is as follows: 'Said Board of Examiners shall be composed of not less than three practicing physicians of known ability and who are graduates of some medical college, recognized by the American Medical Association, and who are residents of the district for which they have been appointed.'

"We contend that this article is unconstitutional because it is in conflict with a provision of the Constitution of the State which prohibits the Legislature from giving preference to any particular sect or school of medicine, for the reason that the only persons authorized to be appointed upon the Board of Medical Examiners are those who ase graduates of some medical school recognized by the American Medical Association, and that this Association was organized and controlled by allopathic physicians, and recognize no graduate or college but allopathic, and these are recognized to the exclusion of *all* other schools, i. e. eclectic, heomopathic, etc."

From this is seen that the Constitution of the State distinctly prohibits discriminative legislation as regards the practice of medicine, and yet the Legislature, which created the present law, violated a constitutional provision. It is difficult to understand how the two may be harmonized.

The Court of Civil Appeals sustained the decision of the lower court, and I am informed that the court in rendering its decision stated with much emphasis that the mere fact of there being no board in the district where I lived was no excuse for not having complied with the law. That I should have applied to some other board, that is to say, that I should have gone to some other district (no matter how remote) and applied for registration.

It is my opinion that at the time the suit was instituted there were no medical boards in the entire State that had organized in accordance with the present law, therefore, according to the rulings of the courts a physician is expected to do an impossible thing, and no allowance is made for the fact that the trouble has been occasioned by the derelictness of the officers of the law. It is clear, however, that in the opinion of the courts of this State, a physician who has not registered in accordance with this law, cannot recover for his services by civil suit, no matter what his professional qualifications may be. *This may be law, but it is certainly not justice.*

Quoting again from the brief: "The courts of New York State in the case of French vs. Gridley, 29 Wendell, 469, and in the case of Bronson vs. Huffman, 7 Hun. 674, have held that a physician could recover for his services although he may not have been licensed to practice as provided by the New York law, which decision seems to us to be consonant with justice and common honesty, and we believe that this view of the law ought to prevail in this State. That the appellant in this case rendered the services set out in this petition to the defendant, and that the services were reasonably worth the amount charged therefor, is admitted, which, being the case, it seems to us that justice and equity demand that he ought to have the right to recover such sum from the defendant."

But, as has been already stated, the honorable court thought otherwise. This case has forcibly recalled to the mind of the writer the story of Bleak House, by Charles Dickens, wherein he describes the suit in Chancery of Jarndyce and Jarndyce, with the lawyers mistily engaged in one of the ten thousand stages of an endless cause, tripping each other up on slippery precedents; groping knee-deep in technicalities and making a pretense of equity with serious faces as players might, and compares the row of solicitors to a long matted well, to the bottom of which you might look in vain for the truth, having before them bills, cross bills, answers, rejoinders, injunctions, affidavits, issues, references to masters, masters' reports, mountains of costly nonsense piled before them.

The design of the law is to protect the rights of citizens and to mete out justice, but the distortions accomplished by clever lawyers in the dexterous wielding of technicalities turns this powerful engine of good to a base purpose, and places a premium upon dishonesty and crime. The teaching of law in schools is apt to inculcate an ambition along this line, as is evidenced by the following statement which emanated from a distinguished professor of law: "Gentlemen, the question which you will be called upon to decide in the practice of your profession, is not whether it is *right*, not whether is it *just*, but *is it law?*

The physician who would refuse to treat a patient unless he received his fee in advance, would be ostracised by the community and considered inhuman, and yet after the service has been rendered the patient may refuse to pay, and through the slippery mazes of the law find an honorable (?) discharge of his obligation. without compensating the physician.

If our legislators would be more careful in their consideration of the laws which they formulate, many of the technicalities would be eliminated, and it would then be possible to obtain *justice* through the processes of *law*. The present medical law is very contradictory in its various clauses. For instance, if a physician has a diploma from a reputable medical college and has presented it for record with the district clerk, he may practice medicine without molestation from the myrmidons of the law and is not criminally liable. Whilst another clause states that if a physician engages in the practice of medicine without having a certificate from an Examining Board, he shall be deemed guilty of a misdemeanor and be subject to the penalty imposed by law.

The present status of the law seems to be, that a physician who has a diploma from a reputable medical college and has presented the same for record, may practice medicine without violating the criminal code, but unless he has obtained a certificate from an Examining Board, he cannot recover for his services by civil suit.

The decision rendered in this case ought to impress upon the profession of this State the defective character of the laws regulating the practice of medicine, and stimulate us to the securing of more just and wholesome regulations which will give to us the same privileges and protection as is accorded other classes of citizens.

